# Quorum Sensing and Expression of Virulence in Pectobacteria

**DOI:** 10.3390/s120303327

**Published:** 2012-03-08

**Authors:** Lee Põllumaa, Tiina Alamäe, Andres Mäe

**Affiliations:** Department of Genetics, Institute of Molecular and Cell Biology, University of Tartu, Riia 23, Tartu 51010, Estonia; E-Mails: lee.pollumaa@ut.ee (L.P.); talamae@ebc.ee (T.A.)

**Keywords:** quorum sensing, N-acylhomoserine lactone, autoinductor 2, virulence, *Pectobacterium*

## Abstract

Quorum sensing (QS) is a population density-dependent regulatory mechanism in which gene expression is coupled to the accumulation of a chemical signaling molecule. QS systems are widespread among the plant soft-rotting bacteria. In *Pectobacterium carotovorum*, at least two QS systems exist being specified by the nature of chemical signals involved. QS in *Pectobacterium carotovorum* uses N-acylhomoserine lactone (AHL) based, as well as autoinducer-2 (AI-2) dependent signaling systems. This review will address the importance of the QS in production of virulence factors and interaction of QS with other regulatory systems in *Pectobacterium carotovorum.*

## 1. Introduction

*Pectobacterium carotovorum* subsp. *carotovorum* (*Pcc*) [ex *Erwinia carotovora* subsp. *carotovora*] and *Pectobacterium atrosepticum* (*Pba*) [ex *Erwinia carotovora* subsp. *atroseptica*] are plant pathogens which cause soft rot in a wide range of plant species, including many crops of economic importance such as vegetables, ornamentals and also the model plant *Arabidopsis*. Pectobacteria are often described as brute-force pathogens because their virulence strategy relies on plant cell-wall degrading enzymes (PCWDE) which disrupt host cell integrity and thus promote rotting [1,2]. Symptom progression depends on aggressiveness of the bacterial strain and susceptibility of the host plant, but also on environmental conditions, among which temperature and humidity are particularly critical [3]. Coordination of virulence factor synthesis is crucial for the pathogenicity. Regulation of the virulence in *Pcc* is coordinated by quorum sensing (QS) [4,5] through a complex set of transcription factors and posttranscriptional regulators [6–13]. A highly complex manner of regulation of PCWDE production may reflect a demand for very precise regulation of certain genes in response to both environmental stimuli and population density. The finely tuned regulation enables a certain group of bacteria to launch a unified coordinated response to environmental stimuli and to accomplish tasks which would be difficult or even impossible to be achieved by a single bacterium.

Though QS plays a key role in regulation of a battery of PCWDE in pectobacteria, it is important to note that it is only one component of an extremely complicated regulatory network that modulates expression of virulence factors. During the infection, PCWDE are not synthesized until the bacterial population density exceeds a certain number [14]. While the QS model proposes that the cells use signal molecules to sense population density, the diffusion sensing model suggests that signal molecules are used by cells to evaluate mass-transfer properties of the environment [15]. According to the latter hypothesis, pectobacteria may use QS to make sure that gene products (e.g., PCWDE) are produced only then if they will have a beneficial effect for the bacterium. One may envisage that accumulation of signal molecules may enable the cells to discriminate between certain environments such as host plant tissue and soil (plant pathogens) or light organ tissue and sea water *Vibrio fischeri* (*V. fischeri*). The PCWDE secreted by pectobacteria are potential activators of the plant response to infection. So, if bacterial population is very small, it would make sense to postpone enzyme production until they are absolutely required [16]. On the other hand, synthesis of PCWDE may also be just a response of pectobacteria to nutrient limitation as the population size increases. In this case, sensing of population increase will trigger transcription of the genes for PCWDE degradation and further growth will be supported by liberation of additional nutrients from the host plant [17]. In this way pathogens might be able to put off expression of PCWDE for as long as possible to prevent PCWDE production until a suitable population density has been achieved at which it can overcome the host defence system.

## 2. AHL-Based Quorum Sensing in Pectobacteria

### 2.1. General Aspects of AHL-Based Quorum Sensing

Quorum sensing was first described as a regulator of bioluminescence in *V. fischeri* and *Vibrio harveyi* (*V. harveyi*) [18,19] in which acyl-homoserine lactons (AHLs) act as signaling molecules. Since then, in many Gram-negative bacteria several kinds of AHLs (called autoinducers) have been identified as signal compounds for this mechanism [20,21]. Bacteria that produce AHLs can respond to a local concentration of signaling molecules, and high population densities (10^10^ cells mL^−1^) foster accumulation of inducing levels of AHLs. The chain length (C4-C18) and the oxidative status of the acyl side chain of AHLs vary and reflect the observed species-specificity of this communication system [22]. QS allows the bacteria to respond to fluctuation in their numbers and enables a synchronous regulation of target genes when living in a community. Clinically important and commercially relevant pathogens often use QS to control various cellular processes [23]. AHLs are central to the regulation and have been shown to control processes as diverse as bioluminescence in marine bacteria *V. fischeri* [24], biofilm formation and virulence in an opportunistic pathogen *Pseudomonas aeruginosa* [25], conjugal transfer of the Ti plasmid in *Agrobacterium tumefaciens* [26,27], production of the exopolysaccharide stewartan acting as a virulence factor in a maize pathogen *Pantoea stewartii* [28,29] and production of carbapenem antibiotics as well as PCWDE in a plant pathogen *Pectobacterium carotovorum* [4,5]. Although the QS systems are used by many plant pathogenic bacteria to regulate the virulence, they are not essential for their survival under laboratory conditions. This indicates that the need for QS is more urgent in the natural habitat, where bacterial population density fluctuates in response to environmental conditions.

The luciferase operon in *V. fischeri* is regulated by two proteins, LuxI (I-protein), which is responsible for the production of the AHL autoinducer, and LuxR (R-protein), which is activated by this autoinducer to increase the transcription from the luciferase operon [24]. Although the LuxR-type proteins have similar structures, their operating mechanisms can be different. So, in contrast to LuxR, a LuxR-type protein EsaR of the plant-pathogen *Pantoea stewartii* [30] binds the target DNA in the absence of AHL and represses the transcription, whereas binding of cognate AHL is thought to be needed for the release of EsaR from the DNA to enable derepression of the target gene [31,32].

The LuxI type proteins are AHL synthases that use S-adenosylmethionine (SAM) to synthesize the homoserine lactone ring, and the acyl chains come from lipid metabolism provided by various acyl-carrier proteins [32,33]. Several studies have revealed a conserved region with a number of specific residues required for LuxI activity. This region comprising residues 25–104 of the 193-aa LuxI polypeptide has been proposed to represent the active site for formation of the amide bond between the acyl group and the amino donor, SAM [34,35]. Acyl chains vary in number of carbons from 4 to 18 and are sufficiently diverse to ensure specific recognition of AHLs by different LuxR-type proteins.

The LuxR type proteins are transcription factors, which upon binding the AHL signal molecule modulate transcription of their target genes [36,37]. While most QS systems conform to this pattern, there are examples of the binding of LuxR-like proteins to the DNA in their native state [30]. The LuxR-type proteins share an end-to-end sequence identity of 18–28%. The AHL-interacting region (66–38 aa) and a DNA binding motif (183–229 aa) are defined by two clusters of stronger sequence conservation [38]. Only after the AHL concentration reaches a critical threshold level corresponding to a certain quorum of the bacteria, the AHL interacts with the cognate R-protein. The LuxR-type proteins act as QS regulators. They distinguish between different AHLs by binding only a cognate AHL suggesting their involvement in mainly intraspecies signaling [39]. In different *Pectobacterium* strains there are examples of LuxR-type proteins which can recognize AHLs with different side chains [40–42]. Many LuxR-type activators, including CarR, LuxR and TraR, the latter being the only crystallized LuxR-type protein so far [37,43,44] form dimers or multimers upon binding the AHLs [42,45]. Originally, the DNA sequence element required for LuxR-AHL binding was called the *lux* operator, but now mostly the *lux* box [46]. The *lux* box is a 20-base pair inverted repeat sequence located upstream of the *lux* operon transcriptional start site and centered at position −42.5. Additionally, both arms of the dyad repeat are required for activation [38].

### 2.2. ExpI/ExpR Quorum Sensing in *Pectobacterium*

Within the *Pectobacterium* genus, a particular bacterial species, and depending on the strain, normally synthesizes only one or two major AHL signals, sometimes along with some minor AHL products. *Pcc* and *Pba* strains have been divided into two classes on the basis of produced AHL. Class I strains, such as *Pcc* EC153 and SCC3193 synthesize predominantly N-3-oxooctanoyl-l-homoserine lactone (3-oxo-C8-AHL), along with lower amount of 3-oxohexanoyl-l-homoserine lactone (3-oxo-C6-AHL). In contrast, class II strains which include *Pcc* ATCC39048 (and its derivatives, e.g., GS101, ATTn10, and MS1), *Pcc* 71, *Pcc* SCC1 and *Pba* SCRI1043 produce predominantly 3-oxo-C6-AHL, whereas little or none of 3-oxo-C8-AHL [47]. In these class I and class II strains of *Pectobacterium*, a single LuxI homologue has been described for each strain being responsible for the production of all AHLs produced by the strain (Table 1). The AHL biosynthetic specificity mainly resides with AHL synthases [47] and availability of the precursors [40,48]. LuxI homologues of different strains have different names. So, the LuxI counterpart in *Pcc* 3193 and *Pba* SCRI1043 is called ExpI [4,49], in *Pcc* ATCC39048 CarI [23], in *Pcc* EC153 AhlI [48] and in *Pcc* 71 AhlI [50] or HslI [6]. Protein alignment revealed that AhlI of *Pcc* 71 (accession no. L40174), ExpI of *Pba* SCRI1043 (accession no. CAG73025) share 93 to 99% identity, whereas the identity of these proteins with respective homologs from *Pcc* SCC3193 (accession no. X80457), *Pcc* EC153 (accession no. DQ093124) and *Pba* CFBP6272 (accession no. AJ580600) is only 70%.

Brader *et al*. [40] have studied the specificity of AHL signaling in *Pcc* and demonstrated the molecular basis for the substrate chain length specificity of the AHL synthase ExpI of *Pcc* SCC1 and SCC3193. The *Pcc* SCC1 and SCC3193 produce 3-oxo-C6-AHL and 3-oxo-C8-AHL as their main autoinducers, respectively. The difference in chain length specificity of AHL production is accompanied by a higher sensitivity toward 3-oxo-C8-AHL in *Pcc* SCC3193 and towards 3-oxo-C6-AHL in *Pcc* SCC1. The type of AHL produced is dependent on corresponding I-protein [33,50]. So, the *expI* mutant of SCC3193 which produces no detectable amount of AHLs will produce either 3-oxo-C6-AHL or 3-oxo-C8-AHL after introduction of the plasmids harboring *expI*_SCC1_ or *expI*_SCC3193_, respectively. Mutagenesis of the AHL synthase gene *expI*_SCC1_ identified amino acid replacements M127T and F69L in ExpISCC1 that altered the chain length of the produced autoinducer—a 3-oxo-C6-HSL producer was converted to a 3-oxo-C8-AHL producer. These substitutions increased the size of a putative acyl moiety binding catalytic pocket creating space for the larger C8 side chain. This observation suggests that acyl chains of different length may be accommodated by coordinated sequence differences in and near the binding pocket of the ExpI protein.

Though the pattern of produced AHLs is mainly dependent on the I-protein, it can also be modulated by external milieu [47]. In *Pcc*, different growth media or growth conditions result either in very complex AHL profile containing more than 10 different AHLs (in case of LB medium) or a very simple one with only few AHLs (in case of M9 minimal medium and potato). It seems that under stringent *in planta* growth, only few AHLs have a role in determining the virulence as indicated by a poor AHL profile of *Pcc* in the host plant [40].

In pectobacteria, the AHL-based QS system relies on a transcriptional regulator protein ExpR which belongs to LuxR family of proteins. ExpR detects the AHL signal and transduces it into cellular response. There are similar inverted repeat elements ranging from 18 to 22 bp, generally called exp-type boxes, associated with the promoters of genes regulated by ExpR-type proteins in different *Pcc* and *Pba* strains [48]. Chatterjee *et al*. [48] demonstrated that several members of *Pectobacterium* subspecies which are otherwise related possess two types of structurally and functionally distinct ExpR proteins. The ExpR proteins of class I strains represented by AY894424 of *Pcc* strain EC153, X80457 of *Pcc* SCC3193 and AY 80600 of *Pba* strain CFBP 6272share over 95% identity with each other. In contrast to that, the ExpR protein (AY894425) of the class II strain *Pcc* 71 shares only ca 60% of homology with ExpR proteins of class I strains [48]. Major differences between the ExpR proteins of these two groups of strains rely in their N-terminal AHL-binding domains. The ExpR proteins of class I strains mostly interact with 3-oxo-C8-AHL whereas those of the class II strains bind mostly 3-oxo-C6-AHL.

In *Pba* SCRI1043, in addition to *luxR* homologue *expR* (renamed as *expR1*) found adjacent to its cognate *luxI* gene, a second *luxR* homologue which has been termed *virR* appears to be present in the genome. Sequence analysis revealed the presence of VirR homologue ExpR2 in *Pcc* strains 71 and SCC3193 [13,52]. Sequence analysis revealed that ExpR2 of *Pcc* strain 71 exhibits a high level of identity (93%) with VirR of SCRI1043. The ExpR2 of SCC3193 shows 94% aa identity (97% similarity) to VirR of *Pba* SCRI1043 [13]. Based on genetic and functional homology, it was concluded that VirR is genetically identical or very similar to ExpR2 [52].

*Pcc* strain ATCC39048 has also a third *luxR* gene homologue called *carR*, which is apparently mediating QS control of carbapenem antibiotic production in this strain [53]. Unlike *expR1* and *virR/expR2*, *carR* functions as an activator which positively regulates carbapenem production in *Pcc* [53].

### 2.3. Role of ExpI/ExpR in Production of PCWDE

The regulation of PCWDE production in *Pcc* and *Pba* is a well-studied example of AHL-dependent gene expression [4,5]. Inactivation of *expI* has led to decreased production of PCWDE and decreased virulence. The simplest way for activation of virulence gene expression by ExpR is its direct binding to promoter regions of the genes to be transcribed. The initial studies with ExpR (renamed as ExpR1) of *Pcc* SCC3193 did not establish that clear role for the AHL receptor protein in PCWDE production. Inactivation on *expR* (renamed as *expR1*) had very little effect on extracellular pectinolytic enzyme production and also on AHL synthesis. However, multiple copies of *expR1* had some inhibitory effect on PCWDE production [54]. ExpR1-like other members of the LuxR family of regulators have a DNA binding domain and they are believed to function as transcription factors. Cui *et al*. documented that in *Pcc* 71 ExpR1 has no direct effect on *pel1* (pectate lyase) or *ohlI* (*luxI* homologue) transcription [50,55]. Interestingly, studies with *Erwinia chrysanthemi* (*E. chr*) ExpI/ExpR system presented a very different picture. Inactivation of *expI* had only little effect on pectinases synthesis in *E. chr* as expression of only two of the pectate lyase genes, *pelA* and *pelB* was decreased. *E. chr expR*^−^ mutants still produced AHLs and pectinases. However, gel shift and DNase I footprintig experiments showed that ExpR protein binds specifically to promoter regions of the five major *pel* genes. The ExpR–DNA band-shift profiles changed in the presence of AHL [56,57]. Based on these results it is clear that operation mode of the QS systems is fundamentally different in these two groups of soft-rotting bacteria.

It has been proposed that there is a connection between QS- and Rsm system-mediated regulation of PCWDE synthesis. The Rsm system involves RsmA, a protein that is able to complex with mRNAs of PCWDE genes to trigger their degradation by RNAses, and a small noncoding *rsmB* sRNA. The *rsmB* sRNA regulates gene expression by binding to RsmA protein impeding its activity [6,50,55,58]. Early studies have revealed a negative regulatory role of AHL on *rsmA*, for example: (1) inactivation of *expI* causes an increase in the level of *rsmA* transcript; (2) addition of exogenous AHL to the *expI*^−^ mutant reduces the level of *rsmA* RNA to that detected in the parent strain and restores the production of PCWDE [10,11]. These observations suggested that at least part of the regulatory role of QS in *Pcc* strains 3193 and 71 is mediated by its effect on *rsmA* expression.

**Figure 1.**
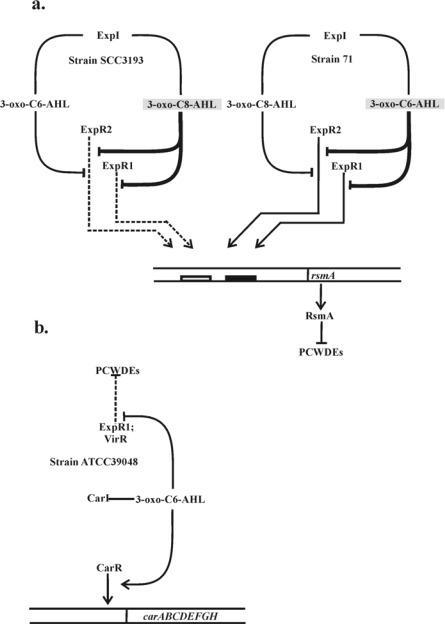
The AHL-mediated quorum sensing-dependent regulation of multiple target genes in *Pectobacterium carotovorum*. (**a**) Regulation of PCWDE production by ExpR1 and ExpR2 in *Pcc* strains SCC3193 (a class I strain) and 71 (a class II strain). (**b**) A schematic model for regulation of carbapenem production in *Pcc* strain ATCC39048 (a class II strain) by CarR in a signal-dependent way. The major AHL species produced by the *Pcc* strains SCC3193 and 71 are shown on gray background and their effects are indicated by bold lines. Inhibitory effects are indicated by ┤ and arrows indicate a stimulatory effect. Not all molecular mechanisms encompassed by this model are fully characterized (indicated on the figure with dashed lines).

The studies by Chatterjee and associates provided further confirmation for the connection between the QS and RsmA protein through discovery that ExpR1 activates the *rsmA* transcription [48,50]. These studies demonstrated that the ExpR1-RsmA system is different from a typical LuxI-LuxR regulatory system since ExpR1, but not the ExpR1-AHL complex activates the transcription of *rsmA*. The ExpR1 protein in *Pcc* 71 was shown to bind *in vitro* the *rsmA* promoter in the absence of AHL and this binding was abolished by the addition of the AHL signal, 3-oxo-C6-AHL (but not 3-oxo-C8-AHL) (Figure 1(a)). In the absence of AHL signals, the receptors ExpR1 and ExpR2 activate the transcription of *rsmA* that encodes a global regulatory RNA-binding protein which reduces the half-life of PCWDE mRNAs. The action of ExpRs is neutralized by their interaction with AHL. In both strains the ExpR1 has strict specificity for the signal molecule: in strain SCC3193 ExpR1 is released from *rsmA* only in the presence of 3-oxo-C8-AHL whereas in strain 71 only in the presence of 3-oxo-C6-AHL. The activator function of ExpR2 is neutralized by both signal molecules. The nucleotide sequence of the ExpR1 binding site was similar to *lux*-box, designated in *Pcc* as the *expR*-box [48]. The fact that ExpR1 acts as an activator only in the absence of the AHL signal explains why it was not previously isolated in genetics screens for QS-dependent regulation of PCWDE production. These observations clearly explain why components of the ExpR1/ExpR2 QS system have opposite effects on PCWDE in *Pcc*.

As a mutation in *expR1* gene in the AHL^+^ background of *Pcc* 71 had almost no effect on levels of PCWDE and RsmA, and level of PCWDE production in an *expR1*^−^ *ahlI*^−^ mutant was higher than that of in *expR1^+^ ahlI*^−^ strain, but still lower than in *Pcc*71, it referred to the possibility that there is more than one functional *expR* gene in *Pcc*71. Support for this suggestion came from the analysis of the whole genome sequence of *Pba* SCRI1043 [49], which disclosed two open reading frames corresponding to AHL binding proteins (putative ExpR homologues).

The data presented by Cui *et al*. [51] confirmed also the presence of another functional *expR* homolog, *expR2*, in the genome of *Pcc*71. Burr *et al*. reported the presence of a second LuxR homolog, VirR, which negatively regulates the production of PCWDE and virulence in the absence of AHL in *Pba* SCRI1043. Based on genetic homology, it was concluded that *virR* is genetically identical or very similar to *expR2* in strain 71. The *virR* (*expR2*) gene appears to be present in a number of *Pectobacterium* strains including *Pcc* ATCC39048, SCC3193, 71 and *Pba* SCRI1093 [52]. Comparative analysis of *Ecc* 71 ExpR2 with previously characterized ExpR1 revealed that they have 62% of identity and 81% of similarity. Both ExpR variants contain autoinducer-binding domain at the N-terminus and helix-turn-helix motifs at the C-terminus [51]. ExpR1 and ExpR2 also differ in their AHL-interaction pattern as ExpR2 only binds 3-oxo-C8-AHL (this can be explained by differences between the AHL binding sites in ExpR1 and ExpR2). Examination of the sequences of ExpR1 and ExpR2 revealed significant differences (46 of 150 residues) between the N-terminal autoinducer-binding domains of these two proteins. This diversity is responsible for significant differences in the binding affinities of ExpR variants for AHL analogs [51].

Gel mobility shift data demonstrated that both, ExpR1 and ExpR2 bind the *rsmA* promoter region. However, the DNA binding properties of these two ExpR variants are different. The ExpR2 binds poorly to the 20-mer expR-box upstream of the *rsmA* which is sufficient for the binding of ExpR1. Thus, in addition to the 20-mer *expR* box, a second 16-mer binding site is necessary for ExpR2-*rsmA* binding (Figure 1(a)). Sequence analysis revealed that the two ExpR variants both contain motifs for DNA binding at the C-termini of the protein and differ by 3 (Leu196Pro, Gly203Asp, and Thr206Ile). out of 56 amino acid residues. The three-residue difference may explain the requirement for the second binding site of ExpR2 in *Pcc* 71 [51].

In *Pcc* 71 ExpI^−^ ExpR2^−^ mutant background reduced level of RsmA and high levels of exoenzymes were detected. This contrasts with almost complete inhibition of exoenzyme production and a high level of RsmA in an ExpI^−^ ExpR1^−^ mutant. These observations establish ExpR2 as the major regulator of exoprotein production and indicate that ExpR1 plays an ancillary role in exoprotein synthesis regulation primarily modulating the level of the RsmA protein [51]. Burr *et al*. [52] demonstrated that *virR* mutation restored the PCWDE production and virulence in the AhlI^−^ mutant of SCRI1043; thus, these phenotypes closely resemble those resulting from inactivation of *expR2* in *Pcc* 71.

However, in *Pcc* SCC3193 ExpR1 and ExpR2 act synergistically to repress the production of PCWDE and thereby also the virulence in the absence of AHL. By inactivating the QS system of SCC3193, that includes the *expI, expR1* and *expR2* genes, it was shown that a triple mutant was able to produce wild-type levels of PCWDE without the addition of any AHLs or macerated plant tissue similarly or even better than the wild-type. The presence of either ExpR1 or ExpR2 in *expI*^−^ mutant background essentially abolished the production of PCWDE in the absence of AHLs. Intriguingly, in *expI*^−^ exp*R1*^−^ double mutant background the transcription of *rsmA* can be prevented with either 3-oxo-C6-AHL or 3-oxo-C8-AHL, leading to subsequent production of PCWDE. In contrast, expression of *rsmA* is abolished only by the addition of 3-oxo-C8-AHL in the *expI*^−^ *expR2*^−^ mutant. These data show that QS controls *rsmA* expression and suggest that QS regulation of PCWDE can be mediated by RsmA. Interestingly, addition of the cognate, but not of the non-cognate AHL to the *expI*^−^ *rsmA*^−^ double mutant resulted in 30% increase in cellulase activity. So, it cannot rule out the possibility that the ExpR proteins of *Pcc* SCC3193 have a dual role by functioning both in an AHL-bound and in a ligand-free states. These results suggest that though the QS control of PCWDE production is largely mediated by RsmA, the cognate 3-oxo-C8-AHL can trigger an additional RsmA-independent pathway to fine tune the PCWDE production.

The ability of ExpR2 to respond to several different AHLs raises a question of its role. Sjöblom *et al*. speculated that ExpR2 may allow interspecies communication between a *Pcc* strain SCC3193 and neighboring bacteria [13]. On the other hand, variation in environmental and physiological conditions may affect the availability of precursors with different acyl chain length in the acyl-ACP pool which may explain the varied profile of AHLs produced by the cells under different growth conditions. ExpR (ExpR2) with wider substrate specificity may help the bacteria to modulate their virulence factors production according to current situation.

An open question remains whether the ExpR1 and ExpR2 (in SCC3193) bind the same promoter region or several possible ExpR binding sites exist in the *rsmA* promoter. In the *rsmA* promoter of *Pcc* 71 two ExpR sites were recently identified and the binding of ExpR to this sites were verified by DNase I footprint analysis [51]. A similar DNA element was identified in the *rsmA* promoter of SCC3193 and also in *Pba* SCRI1043, but binding of either ExpR to this site was not verified.

RsmA plays at least partially a role in inhibition of exoenzyme production at elevated temperatures. The production of PCWDE is reduced when *Pcc* 71 is cultured at 34.5 °C compared to 28 °C and this trend was correlated with reduction in the concentration of oxo-C6-HSL signal. Decrease of AHL concentration enhanced the *rsmA* expression. It was observed that levels of the *rsmA* transcript and the RsmA protein were increased at elevated temperature, suggesting a potential link between the thermoregulation and the Rsm system. The elevated levels of RsmA at 34.5 °C promote RNA decay and consequently reduce the PCWDE production and virulence. One exception is strain EC153 that produces more AHL at 34.5 °C than at 28 °C, and this contrasts with the responses of other *Pcc* strains, including Ecc71 [59].

The fact that *Pcc* strains lacking the QS system (*expI*^−^ *expR1*^−^ and *virR*^−^/*expR2*^−^ triple mutant) are still able to grow and macerate plant tissue similarly to wild-type cells under laboratory conditions indicates that the QS system is biologically relevant mainly in natural habitat, where densities and the composition of bacterial populations fluctuate in response to environmental cues. An ecological study would be essential to elucidate the significance of QS in success of *Pcc* in the environment [1,60,61].

### 2.4. Role of Quorum Sensing in the Production of other Virulence Factors

For a long time only little was known about the extent of QS control within a single microorganism. So, previous studies showed that AHL is required in a *Pc* subspecies for the expression of PCWDE genes [4,6,54]. To identify new virulence genes in *Pba* that may be controlled by QS, Pemberton *et al*. [62] carried out a genome-wide screen using transposon mutagenesis. They isolated seven new genes controlled by QS signal molecule AHL and demonstrated that at least one of these genes, *nip*, is involved in pathogenesis on potato and can induce necrosis in tobacco.

Corbett *et al*. [63] used the proteomic technique of two-dimentional difference gel electrophoresis analysis of the wild-type and *expI*^−^ negative mutant of *Pba* SCRI1043 to identify QS-dependent proteins in the secretome. This study identified three novel QS-dependent secreted enzymes: Svx, a putative proteoglycan hydrolase and a putative cellulase, in addition to previously known QS-dependent secreted PCWDE. Although such proteomic analysis provides valuable information in identifying and comparing secreted proteins from wild-type pectobacteria and defined mutants, many proteins may be missed because they are poorly soluble, present in very low amount or expressed only *in planta*.

Analysis of the full genome sequence of *Pba* SCRI1043 has revealed several other putative virulence factors [49]. Importantly, for many years the role of QS in pathogenesis with regard to PCWDE regulation was studied intensively only *in vitro*. In 2008, a whole genome microarray approach was used by Liu *et al*. for an *expI*^−^ mutant *Pba*1043 grown *in planta* to determine global effects of QS on gene regulation during potato infection, with particular emphasis on the relationship between PCWDE production and possible stealth mechanisms [2]. According to transcriptomic analysis by Liu *et al*., approximately 26% of all *Pba* genes are QS-regulated, suggesting that QS may more strongly contribute to pathogenesis than previously thought. Given that many important secreted PCWDE are produced by *Pectobacterium* in a QS-dependent fashion, it is also important to consider how these secreted proteins are moved out of the bacterial cell. Most interestingly, QS in *Pba* was shown also to modulate type I (T1SS) and type II (T2SS) secretion systems which are considered “accessory virulence factors” responsible for the delivery of PCWDE. T1SS and T2SS genes exhibited reduced transcription in the *expI* mutant. It was also observed that type III (T3SS) secretion system structural, putative effector, and helper genes are also downregulated in the *expI* mutant. The finding that QS regulates the entire T3SS indicates that coordinated physical (PCWDE) and stealth (T3SS) attack may be necessary for successful infection and disease development. The QS regulon also included a novel Type VI secretion system (T6SS), its predicted substrates Hcp and VgrG and over 70 known or putative regulators, some of which have been demonstrated to control pathogenicity. The complexity of QS circuit indicates that it is governing multiple other regulators that in turn precisely regulate the destructive arsenal of PCWDE during the infection [2].

### 2.5. Role of Quorum Sensing in Carbapenem Production

A few strains of *Pcc* from geographically diverse sources produce low levels of a β-lactam antibiotic, carbapenem [64]. The genes for carbapenem production are clustered in an operon of eight genes (*carA-H*) [65] and are controlled in a QS manner in response to accumulation of AHL [5,66,67]. The *car* gene cluster of *Pcc* ATCC39048 was the first among *Pcc* strains to be sequenced and both functionally and transcriptionally defined [53]. QS in *Pcc* ATCC39048 involves the production of the diffusible signaling molecule 3-oxo-C6-AHL, which is synthesized by a LuxI family member CarI (an ExpI homologue). CarR, a member of the LuxR family transcription factors, is a DNA-binding transcriptional activator of *carA–H* that functions in the presence of AHL. Interestingly, cryptic *car* gene clusters have been identified in a number of *Pectobacterium* strains [64]. Antibiotic production can be induced in some of these strains by the provision of *carR* from ATCC39048 in trans, suggesting that lack of carbapenem production in these bacteria is due to defects in their CarR proteins or defects in CarR production [64]. The CarR–AHL complex binds to the *carA* promoter in the *carR–A* intergenic region (Figure 1(b)). QS depends on a diffusible signal molecule 3-oxo-C6-AHL produced by CarI synthase. 3-oxo-C6-AHL binds to the regulatory protein CarR. The active CarR-AHL complex binds directly to the *carA* promoter and activates transcription of the *carA-H* operon. This strain has two additional *luxR* homologues, *expR* (renamed as *expR1*) and *virR* which are involved only in regulation of PCWDE production. In *rsmA* promoter the expR box (filled rectangle) and the 2nd binding site for ExpR1 and ExpR2 proteins (empty rectangle) are designated. No *lux* box-like elements have been identified in this region [41,68]. The CarR-mediated AHL-dependent expression of the carbapenem operon results not only in production of carbapenem antibiotic encoded by *carA-E* genes and also expression of carbapenem resistance functions encoded by *carFG* [68]. Disruption of *carI* and/or *carR* abolishes carbapenem production and transcription from the QS-dependent *carA* promoter [68]. However, inactivation of either *expR* or *virR* has no obvious effect on carbapenem production, either in the presence or absence of a functional *carI* gene. Therefore, it appears that CarR is the only LuxR homologue regulating carbapenem production in ATCC39084. It should be noted that neither CarR nor CarR-AHL complex controls PCWDE production (Figure 1(b)). Like many LuxI/LuxR systems, the *Pcc carI* gene has an overlap with the *luxR* gene that encodes a second *luxR* gene *expR* (renamed as *expR1*) involved in regulation of PCWDE [23]. It is clear that carbapenem-producing *Pcc* strains have a single AHL synthase gene CarI, and two different LuxR homologues that recognize the same AHL but control the expression of different genes [41]. Taken together these data clearly demonstrate that *Pcc* has two different LuxR homologues for the control of carbapenem and PCWDE production (Figure 1(b)) [65,69,70]. Coregulation of these two processes, synthesis of the antibiotic and PCWDE via AHL-dependent QS implies that a high cell population density of pectobacteria, having generated a substantial food resource through degradation of plant cell wall material will also promote carbapenem production to eliminate the competing bacteria from the infection site *in planta*.

### 2.6. Environmental Impact of AHL Stability in Quorum Sensing

An earlier report showed that AHL levels rise during the *log* phase of growth and then decline during the stationary phase when bacteria are cultured in rich synthetic media [64]. Under alkaline conditions, AHLs are hydrolyzed and thereby are unable to function as signal molecules for LuxR-type receptor proteins. AHL become unstable over a narrow pH range (pH from 7 to 8). The sensitivity of AHL to extreme pH (pH of >8.2) may be important during infection *in planta*. So, one of the first recognizable responses of plants to *Pectobacterium* infection is the pH increase of the apoplastic fluid around the infection site [71]. This is achieved by activating a very rapid H^+^ influx into plant cells during the early stages of infection (prior to any signs of tissue maceration) which increases the intercellular pH from 6.4 to 8.3 [72]. The most likely mechanisms to break down AHLs are either hydrolysis of the amide bond between the lactone ring and the acyl chain or hydrolysis of the ester bond within the lactone ring. The latter bond is more labile and delactonization of AHL has been shown to occur *in vitro* at pH 12 [73]. Instability of AHL also increases at high temperature. Although in *Pcc* strain ATTn10 grown at 37 °C *carI* transcription is not affected, the concentration of 3-oxo-C6-AHL is reduced below the amount shown to be required for carbapenem production induction. So, degradation of 3-oxo-C6-AHL at 37 °C may be responsible for the effect of temperature on carbapenem production [68].

Oxygen availability also affects AHL stability. This effect can probably be explained by different pH values of the growth environment resulting from either aerobic or anaerobic metabolism. Aerobic metabolism in complex medium (e.g., L-broth) is known to result in alkalinization of the environment due to the preferential use of weak acids as carbon sources which explains degradation of AHL in stationary phase cultures [74]. Anaerobic metabolism does the opposite, and results in net production of weak acids that may account for the persistence of AHL during stationary phase, and explain why soft rot occurs much more readily when oxygen is limiting [3].

Recently, many different bacteria belonging to various genera were reported to express AHL-degrading activity [75]. Two groups of AHL-degrading enzymes have been identified so far: AHL lactonases [76] and AHL acylases (AiiA) [77]. AHL lactonases hydrolyze the lactone ring in the homoserine moiety of AHLs without affecting the rest of the molecule structure. AiiA from *Bacillus* sp. 24B1 was one of the first AHL lactonases characterized [76]. During recent years more bacteria possessing AHL lactonase activity have been described [37,78,79]. AHL acylases hydrolyze the amide bond between the acyl side chain and the homoserine lactone in the AHL molecule [80]. To the present, nine AHL acylases from various groups of bacteria have been reported and five of them described in detail [77,81,82]. Most of AHL-utilizing bacterial strains were reported as soil or rhizosphere inhabitants [83,84]. The ability to inactivate AHLs might be useful in controlling virulence of many plant pathogenic bacteria [85–87].

The data reviewed here clearly demonstrate that loss of bacterial communication system does not always result from mutational inactivation of AHL synthase (*expI*), but may also be caused by enzymatic or nonenzymatic degradation of AHL under certain environmental conditions.

## 3. LuxS/AI-2-Based Quorum Sensing in Pectobacteria

### 3.1. General Aspects of LuxS/AI-2 Quorum Sensing

A different QS sensing system has been described in several bacteria. This system is based on AI-2 and it was first identified in a Gram-negative bacterium *V. harveyii* [88]. In this QS system, the first signal molecule identified to bind the AI-2 receptor was a furanosyl borate diester. The synthesis of AI-2 is dependent on the enzyme LuxS [89]. In *V. harveyii* at high cell density, AI-2 binds LuxP (a receptor protein), initiating a dephosphorylation cascade which results in dephosphorylation and inactivation of the response regulator LuxO; inactivation of LuxO allows expression of LuxR_Vh_ which in turn activates the expression of the bioluminescence operon [90–92]. Numerous Gram-negative and Gram-positive bacteria in addition to *V. harveyii* have a *luxS* homologue and synthesize compounds capable of activating the AI-2 biosensor [91].

LuxS protein, the product of the *luxS* gene which is widely conserved throughout the bacterial kingdom, is responsible for AI-2 biosynthesis. LuxS synthesizes 4,5-dihydroxy-2,3-pentanedione (DPD), which undergoes spontaneous rearrangements to form a variety of DPD derivatives that interconvert comprising the AI-2 pool. Cyclization of DPD generates compounds (2*R*,4*S*)-and (2*S*,4*S*)-2,4-dihydroxy-2-methyldihydrofuranone *R*- and *S*-DHMF, respectively and boronoate ester formation from DPD occurs if enough borate is present in solution. Because DPD exists in equilibrium with other chemical species in the solution, AI-2 is actually not only a furanosyl borate diester, but this term is used to collectively designate multiple chemical derivatives of DPD [91]. AI-2 responses in different bacterial species can be triggered by different members of the AI-2 pool. Because the chemical nature of the active signaling molecule from this pool varies between the species [92], it is unsurprising that the nature of the AI-2 receptor for these signals is also variable. To date, only three proteins that bind AI-2 signaling components have been characterized [93–95].

The biosynthetic pathway of AI-2 has disclosed a metabolic role for LuxS, involvement in S-adenosylmethionine-utilization pathway [96]. LuxS converts S-ribosylhomocysteine (produced by detoxification of 5-adenosylhomocysteine) to homocysteine (which is recycled back to methionine) and AI-2. Since LuxS has a metabolic role, it is currently unclear how many of the phenotypes described for *luxS*^−^ mutant actually result from a signaling defect due to the absence of AI-2, and how many are caused by hampered methionine recycling.

### 3.2. LuxS/AI-2-Based Quorum Sensing in *Pectobacterium carotovorum*

A *luxS* homologue from a *Pectobacterium* was first reported in a derivative of *Pcc* ATTn10 and in *Pba* SCRI1043 [97,98]. Then, Coulthurst *et al*. [99] used the proteomic approach to identify LuxS-dependent proteins in the secretome of *Pcc* ATTn10 and *Pba* SCRI1043. Production of secreted virulence factors, particularly a multitude of secreted pectinases and other PCWDE was studied. Protein spots corresponding to pectate lyases, cellulase and protease were all present in reduced amounts in SCC2 (*luxS*^−^ mutant) if compared with the wild-type *Pcc* ATTn10. Specifically, the proteins with expression affected in SCC2 were identified as cellulase V (CelV), the major cellulase in *Pcc* and an important virulence factor [97,100], and the secreted metalloprotease, protease W (PrtW), which participates in disease progression [101]. Also, decreased expression was detected for pectate lyase I, II and III (PelA, B and C) isoforms [49,102]. Pectinases are primary virulence determinants of *Pc* and pectate lyases are the most important among the pectinases [103]. The corresponding secreted enzyme activities were also measured for the wild-type *Pba* SCRI1043 and the *luxS*^−^ mutant, SCC10 grown to stationary phase in phosphate minimal medium. Similarly to *Pcc* ATTn10, activities of secreted pectate lyase, cellulase and protease were reduced in the *luxS*^−^ mutant of *Pba* SCRI0143 compared to the wild-type. These results demonstrate that full production of secreted virulence factors pectate lyase, cellulase and protease is dependent on functional *luxS* gene in both *Pba* SCRI1043 and *Pcc* ATTn10, at least under the conditions applied.

The *luxS*^−^ mutants of *Pba* SCRI1043 and SCC10 exhibited modestly decreased swimming motility compared to the wild-type. *luxS* mutants of several other bacteria, including *E. coli* were also affected in motility [104,105]. In *Pectobacterium*, motility enhances the invasion and infection of potato plants [3,106] suggesting that SCC10 may be disadvantaged compared with the wild-type during the initial stages of natural infection.

Reduced level of secreted enzymes in the *luxS*^−^ mutant and reduced motility of the mutant, if reproduced *in planta*, are expected to result in decreased virulence. However, in SCC10 (a *luxS*^−^ mutant) the observed decrease in motility and secreted enzyme production did not translate into a significant decrease in virulence according to the test models applied. In *Pcc* ATTn10, a potato tuber rotting assay was used to compare the effect of *luxS* mutation on virulence. SCC2 (a *luxS*^−^ mutant) exhibited reduced virulence if compared with the wild-type in this test. By contrast, although SCC10 exhibited a slightly lower mean rot than the wild-type *Pba* SCRI1043 in a tuber rotting test, the difference was not statistically significant. The virulence of SCC10 was also compared with that of the wild-type using a potato stem infection assay [63]. SCC10 failed to show a statistically significant reduction in lesion formation over the length of the assay compared with wild-type *Pba* SCRI1043. It is likely that the impact of *luxS* inactivation is dependent on environmental conditions and the nature of the assay used. The reduction in motility of the strain may not have a significant impact in these assays because the bacterial cells were injected directly into the plant tissue, overcoming the need to gain access to the interior of the plant during a ‘real-life’ infection.

In *Pcc* strain SCC3193 inactivation of the *luxS_Pcc_* gene is sufficient to partly repress the production of pectinolytic enzymes during early exponential growth phase (6–8 h). These observations and the pattern of AI-2 production demonstrate that there is a correlation between AI-2 level and production of pectinolytic enzymes. Indeed, the maximum difference in pectinolytic enzyme production between the SCC3193 and *luxS*^−^*_Pcc_* strains coincide with the maximum level of AI-2 in the parental strain SCC3193 which is in the end of exponential growth phase. The fact that *luxS*^−^*_Pcc_* mutant had an impaired, but not abolished pectinolytic enzyme production capacity, suggests that AI-2 was not solely required for, but rather to contribute in maximization of pectinolytic enzymes production at low cell densities [107]. It should be noted that receptors for AI-2 have not yet been identified in *Pectobacterium* strains.

The dramatic consequence of the absence of the AHL type QS signal molecules is reduction of the maceration capacity of *Pcc* [4,5]. The defect in AI-2 production in the *luxS*^−^*_Pcc_* of SCC3193 caused 50% less tissue maceration in the potato tuber test already by first 36 h after the inoculation. The reduced maceration of plant tissue in case of *luxS*^−^*_Pcc_* mutant is not related to alteration of the ability of the mutant to synthesize AHL or grow *in planta*, but rather to the defect in AI-2 production. In agreement with this, the *luxS*^−^*_Pcc_* mutant was able to cause consequent maceration of plant tissue, and by 72 h of the assay no significant differences in tissue maceration between the parental and *luxS_Pcc_* mutant strain were detected [107].

This observation provides evidence for the view that in QS regulation in *Pcc* during later stages of plant infection, the accumulation of AHL signal is sufficient for efficient PCWDE production even in the absence of AI-2 signal. Brader *et al*. [40] have shown that *Pcc* strain SCC3193 produces at least six different AHLs depending on growth conditions. It is tempting to speculate that the roles of AHL species and AI-2 type QS signals may vary at different stages of infection and in different host plants. However, regulation of PCWDE production by two QS systems is certainly clearly different. The one comprising ExpI/AHL, affects PCWDE production through modulation of the *rsmA* expression [10,11]. The other regulatory system comprising LuxS/AI-2 controls PCWDE production by a still unknown mechanism.

It was of interest to determine whether a link can be detected between the *luxS/*AI-2 and the AHL QS sensing systems of *Pcc* and *Pba*. Production of the QS signal molecule AHL throughout the growth was found to be unaffected in both SCC2 and SCC10 mutants compared with their respective wild-type strains [97]. Similarly, production of AI-2 was not affected in the *carI* mutant of *Pcc* ATTn10, strain JBC1, which cannot produce AHL [74]. The production of AHL and AI-2 signal molecules also does not depend on each other in SCC3193 [107].

The mechanism(s) by which *luxS* is ‘regulating’ these phenotypes remain to be determined and several possibilities exist. AI-2 may act as an extracellular signaling molecule. Such a signaling role could involve reporting on the bacterial population density (*i.e*., quorum sensing) as proposed previously [89], but it may also involve sensing of environmental conditions, presence and metabolic status of other cells. It is also possible that AI-2 could act as an intracellular regulatory molecule. Finally, any phenotype, including alteration of the abundance of a given protein in a *luxS* mutant could be the indirect result of a metabolic perturbation due to the loss of LuxS activity in the activated methyl cycle [108]. This final possibility could be excluded by complementation of *luxS*-dependent phenotypes with the addition of synthetic AI-2. The effect of a *luxS* mutation could be mediated differently for different proteins and the molecular basis of *luxS*-dependent regulation in *Pectobacterium carotovorum* remains to be determined.

## 4. Concluding Remarks

Many Gram-negative plant pathogenic bacteria use some variant of QS system, either the ExpR-I type or the LuxS/AI-2 type or both to assess the environment and regulate production of virulence factors. Initially, regulation through ExpR-I type QS system was perceived as a relatively simple model involving an AHL synthase, an AHL signal and an ExpR-type regulator activating specific genes at a high cell density. This model is valid only for carbapenem production regulation by AHL/CarR. The overall picture for virulence regulation is far more complex. So, the AHL/ExpR QS system of *Pcc* governing the production of PCWDE feeds into a post-transcriptional regulatory system which monitor the pool size of RsmA protein which in turn modulates the stability of PCWDE mRNAs. The *LuxS*/AI-2 type QS plays a strain-dependent role in virulence of different *Pectobacterium* strains. The fact that *luxS*^−^ mutant has impaired but not abolished pectinolytic enzyme production suggests that AI-2 does not solely determine but rather contribute to maximize PCWDE production during the infection. The advantage of having two different QS systems is still not entirely clear. It can be that with the aid of two different QS systems, the genes can be regulated also at low cell population densities and under diverse environmental conditions, allowing fine-tuning of QS-regulated traits. A rich diversity of chemical structures among QS signaling molecules used by a pathogen most probably enables population density-dependent modulation of virulence gene expression even under conditions when certain types of signal molecules will be degraded.

## Figures and Tables

**Table 1.** AHL quorum sensing systems of class I and class II pectobacteria.

**Strain**	**Major AHL**	**LuxI/R homologue(s)**	**QS regulated phenotype(s)**	**Reference**

**Class I strains**				

*Pcc* SCC3193	3-oxo-C8-AHL	ExpI/ExpR1/ExpR2	production of PCWDE; virulence	[4,13]
	3-oxo-C6-AHL			
*Pcc* EC153	3-oxo-C8-AHL	AhlI/ExpR	production of PCWDE; virulence	[48,49]

**Class II strains**				

*Pcc* 71	3-oxo-C6-AHL	AhlI/ExpR1/ExpR2	production of PCWDE; virulence	
	3-oxo-C8-AHL			[50–52]
*Pcc* SCRI193	3-oxo-C6-AHL	ExpI/ExpR1/ExpR2	production of PCWDE; virulence	[5]
*Pcc* ATCC390048	3-oxo-C6-AHL	CarI/CarR ExpR1/VirR	production of carbapenem and PCWDE; virulence	[52]
*Pba* SCRI1043	3-oxo-C6-AHL	ExpI/ExpR/VirR	production of PCWDE, Nip	[49,52]
